# Acute Myasthenia Crisis: A Critical Emergency Department Differential

**DOI:** 10.7759/cureus.9760

**Published:** 2020-08-15

**Authors:** Christopher Hogan, Jenny Lee, Bryan C Sleigh, Paul R Banerjee, Latha Ganti

**Affiliations:** 1 Emergency Department, Coliseum Medical Centers, Macon, USA; 2 Emergency Mecicine, Brown University, Providence, USA; 3 Emergency Medicine, Mercer University School of Medicine, Macon, USA; 4 Emergency Medicine, University of Central Florida, Orlando, USA; 5 Emergency Medicine, University of Central Florida College of Medicine/Hospital Corporation of America Healthcare Graduate Medical Education Consortium of Greater Orlando, Orlando, USA; 6 Emergency Medicine, Envision Physician Services, Nashville, USA; 7 Emergency Medical Services, Polk County Fire Rescue, Bartow, USA

**Keywords:** myasthenia gravis

## Abstract

Myasthenia gravis (MG) is the most common autoimmune disorder of the neuromuscular junction (NMJ). It is caused by autoantibodies blocking acetylcholine receptors (AChRs) or structural receptors of the NMJ: agrin, LRP4, and MuSK. These antibodies can block, change, or destroy AChRs or structural proteins of the NMJ, preventing the binding of ACh and therefore, muscle contractions. This molecular dysfunction can manifest as any of the following symptoms: ptosis, diplopia, bulbar dysfunction, or impaired vision in bright light. Symptoms fluctuate in severity throughout the day and with prolonged use of respective muscles. Typical treatment for mild cases is acetylcholinesterase inhibition combined with an immunosuppressor.

Myasthenia crisis results from the exacerbation of the aforementioned symptoms and requires intubation for respiratory support. Intensive care along with intensified immunosuppressive treatments and constant monitoring are recommended. We present the case of a 76-year-old man arriving to the emergency department (ED) with symptoms of fatigue and dysphagia, diagnosed as acute myasthenia crisis. Here, we highlight the symptoms of MG, acute myasthenia crisis, and the critical measures that need to be taken.

## Introduction

Myasthenia gravis (MG) is the most common autoimmune neuromuscular junction (NMJ) disorder, affecting about 20 per 100,000 people in the US [1]. Autoimmune MG is caused by autoantibodies directed against NMJ proteins. In healthy neuromuscular transmission, the motor nerve releases acetylcholine (ACh) which binds to pentameric acetylcholine receptors (AChRs). Another transmission route begins with the release of agrin from the motor nerve, which interacts with low-density lipoprotein receptor related protein 4 (LRP4), thereby activating muscle-specific tyrosine kinase (MuSK), which clusters AChRs at the endplate. Although less common, autoantibodies may prevent ACh from binding to these primary structural proteins of the NMJ: agrin, LRP4, and MuSK. The main AChR antibodies are immunoglobin (Ig)G1 and IgG3, which disrupt NMJ transmission via complement-mediated activation of membrane attack complex and destruction of the postsynaptic membrane; cross-linking AChRs; or direct blocking of the ACh binding site on the AChR. MuSK antibodies, primarily IgG4, inhibit MuSK from clustering AChRs at the endplate and are highly pathogenic [1].

Common symptoms of MG include ptosis, diplopia, bulbar dysfunction, and impaired vision in bright light. Symptoms fluctuate in severity throughout the day and with prolonged use of respective muscles. [1] Treatment usually consists of acetylcholinesterase inhibition with an immunosuppressor, most frequently azathioprine [2]. Myasthenia crisis is life-threatening exacerbation of MG muscle weakness, requiring intubation for respiratory support [3]. Along with intensive care and constant monitoring, intravenous immunoglobulin (IVIG) and plasma exchange are recommended for rapid effects within two to five days. If no improvements are exhibited, high-dose corticosteroids should be administered; otherwise, immunosuppressive treatments should be intensified until symptoms subside. Here, we present the case of a 76-year-old man with symptoms of fatigue and difficulty swallowing, diagnosed as acute myasthenia crisis.

## Case presentation

Emergency department presentation

A 76-year-old male patient arrived at the emergency department (ED) as a transfer from an outside hospital. The patient was awake and alert but complained of fatigue and difficulty swallowing. His vital signs were normal. Upon physical examination, the patient had obvious bilateral ptosis of the eyes and diminished chest movement with respiration. Negative inspiratory force revealed an initial reading of 18 cm/H_2_O and a subsequent reading five minutes later of 15 cm/H_2_O. The patient and his family were informed of the patient’s impending respiratory failure. They agreed to the treatment plan of intubating the patient in order to protect the airway and provide ventilatory support while awaiting definitive care. He was then admitted to the intensive care unit (ICU) where his endotracheal tube was removed three days later without complication. The patient was awake and alert at the time of discharge, during which he provided further pertinent past medical history.

History of present illness

The patient presented signs and symptoms of a urinary tract infection (UTI) at a local urgent care center 24 hours before his arrival at the ED. He stated he was experiencing urgent and frequent dysuria, and lower abdominal pain. He explained that the urgent care was exceedingly busy, so the provider quickly assessed him. He was given an intramuscular injection of ceftriaxone, and discharged home with a prescription for ciprofloxacin 500 mg twice a day for seven days. The provider was treating for potential multidrug-resistant gram-negative organisms due to the patient’s previous stay in the hospital five months prior. About 15 hours after his antibiotic injection, the patient was awakened at home around 0300 by his UTI symptoms so he took the oral ciprofloxacin. He was symptom free at breakfast the next morning, but by lunchtime, he was unable to swallow his food. He recalled feeling this prior to his first acute myasthenia crisis five months ago, and immediately called his neurologist. The neurologist advised him to call emergency medical services (EMS) and get to the closest emergency room. The patient and his wife were concerned because during the previous episode, he was worked up for a differential diagnosis. Due to a prolonged workup with a differential diagnosis excluding myasthenia crisis, he felt he was intubated late.

Past medical and family history

The patient had first been diagnosed with MG five months ago when he exhibited symptoms of difficulty swallowing and bilateral ptosis. He was worked up for a possible cerebrovascular accident and was intubated due to poor respiratory effort. He stayed in the hospital for several days before being discharged with the diagnosis of MG. He was prescribed mestinon 15 mg by mouth every six hours, prednisone, doxazosin, and a statin at that time. He had no past medical history up to that point and no allergies. He denied recreational drug use and smoking. He endorsed drinking alcohol socially. His family history was relevant as his father was diagnosed with MG prior to his death due to lung cancer.

## Discussion

**Figure 1:**
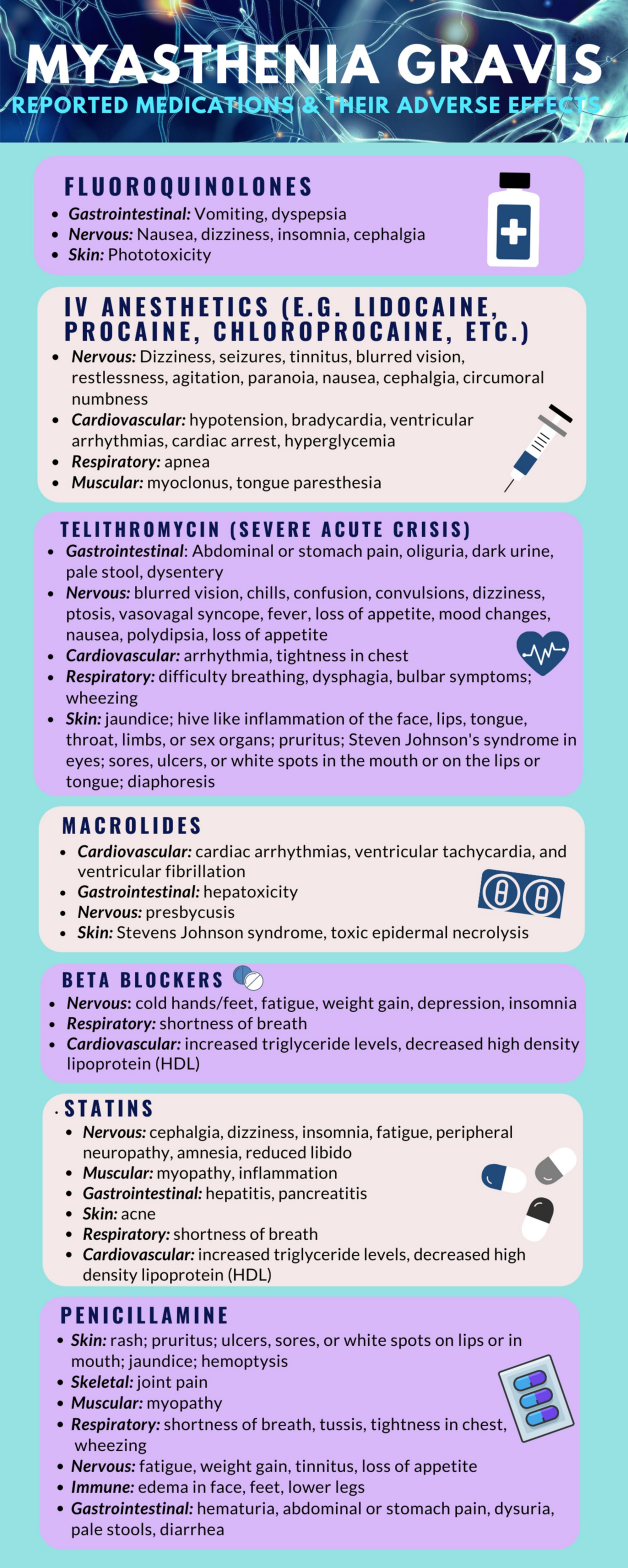
Medications that can cause adverse events in myasthenia gravis

MG is an autoimmune disorder characterized by progressive muscle fatigue with repeated used. In an overwhelming majority of patients, this is caused by autoantibodies to the AChRs on the postsynaptic nerve terminal. Approximately 80%-90% have detectable antibodies to AChRs in serum. Nonetheless, some patients have antibodies to the transmembrane component MuSK. Up to 20% of patients can be seronegative on standard assay, 31%-41% of whom have autoantibodies against MuSK [4]. Other associations are thymoma and thymic hyperplasia, which are seen in 10%-30% and 80%-90% of patients, respectively [5]. Two drug classes have established adverse effects in this patient population, namely aminoglycosides and neuromuscular blocking agents. Other examples of medications that have been reported to have adverse effects are listed in Figure 1.

The list in Figure 1 is not intended to be all inclusive, rather it is meant to illustrate the breadth and varying mechanisms of medications that may exacerbate an acute myasthenia crisis. A general rule of thumb is to assume that any medication may worsen the patient’s condition. When administering a new medication to a known MG patient, it is prudent to observe for symptoms and advise the patient to be vigilant with self-monitoring once discharged. Chronic treatment for MG might include prednisone, azathioprine, or mycophenolate. A patient who presents as taking immunosuppressant therapy should be probed to identify the condition for which it is intending to treat. A thorough history and physical can put MG on the differential.

The initial treatment for mild to moderate exacerbations is an acetylcholinesterase inhibitor, such as pyridostigmine and neostigmine. These drugs inhibit the enzyme responsible for catalyzing the degradation of AChRs in the synaptic cleft. This leads to increased ACh competing for available receptors on the postsynaptic terminal, thereby enabling muscle contractions and increasing muscle strength.

Side effects of treatment with acetylcholinesterase inhibitors can cause cholinergic crisis. Although exceedingly rare, this can be mitigated with anticholinergic therapy, such as glycopyrrolate and hyoscyamine [2]. Patients who experience severe MG should be treated with plasma exchange or IVIG [6].

Patients with MG can present with acute weakness either because they have too much medication (resulting in a cholinergic crisis) or because of an exacerbation of their myasthenia, usually secondary to an acute illness or stress (myasthenic crisis), which results in not enough anticholinesterase inhibitor. It is not always possible to distinguish the two types of crises based on physical examination, but prompt ED management is paramount [7].

## Conclusions

MG and its associated crises are not common ED presentations, but it is imperative to recognize them, as the patient can otherwise go into respiratory failure. Physical examination alone may not differentiate the cause for the respiratory distress; thus, the clinician needs to take a good history in addition to maintaining a high index of suspicion.
